# Research on a Defecation Pre-Warning Algorithm for the Disabled Elderly Based on a Semi-Supervised Generative Adversarial Network

**DOI:** 10.3390/s22176704

**Published:** 2022-09-05

**Authors:** Yanbiao Zou, Shenghong Wu, Tie Zhang, Yuanhang Yang

**Affiliations:** School of Mechanical and Automotive Engineering, South China University of Technology, Guangzhou 510641, China

**Keywords:** generative adversarial network, bowel sounds, disabled elderly, defecation pre-warning

## Abstract

The elderly population in China is continuously increasing, and the disabled account for a large proportion of the elderly population. An effective solution is urgently needed for incontinence among disabled elderly people. Compared with disposable adult diapers, artificial sphincter implantation and medication for incontinence, the defecation pre-warning method is more flexible and convenient. However, due to the complex human physiology and individual differences, its development is limited. Based on the aging trend of the population and clinical needs, this paper proposes a bowel sound acquisition system and a defecation pre-warning method and system based on a semi-supervised generative adversarial network. A network model was established to predict defecation using bowel sounds. The experimental results show that the proposed method can effectively classify bowel sounds with or without defecation tendency, and the accuracy reached 94.4%.

## 1. Introduction

After entering the 21st century, the problem of aging population in the world is becoming increasingly prominent. Studies show that, by 2050, the number of people over 80 years old in the world will increase rapidly from 69 million in 2010 to 379 million [1]. According to the data released by the seventh national Census, one key indicator is that the number of elderly people has hit a new high. Compared with 2010, the population over the age of 65 in China has increased from 8.9% to 13.5% in 2020, which is 19.064 million, indicating that China has entered a serious aging stage [2].

Disabled seniors are those who have lost the ability to take care of themselves. Incontinence is a painful experience for the elderly. First of all, complications, such as urine leakage [3] odor and red skin [4], caused by incontinence will increase the physical pain of patients. Second, incontinence will cause patients to defecate without knowing or at an inconvenient time, bringing embarrassment or even fear to patients and seriously affecting the mental health of the elderly [5].

For the families of patients, incontinence will bring many difficulties to the nursing work of the disabled elderly and increase the economic burden of the family. In the near future, how to properly take care of the disabled elderly is a problem that almost every family will face. For the above reasons, the incontinence of the disabled elderly has become a problem that needs to attract the attention of society as a whole, and a simple and effective solution is urgently needed.

With the improvement of living standards and the development of medical technology, researchers have proposed some measures to solve the incontinence problem of disabled elderly, which can be roughly divided into the following three methods.

1. Disposable diapers and incontinence pads are often the first choice for incontinence patients. The proper use and timely replacement of incontinence pads can minimize the risk of skin odor and leakage and make life more comfortable for the disabled elderly. Disposable diapers and incontinence pads were first used in nursing homes in the United States in the 1980s [6], followed by countries, such as Ireland and the United Kingdom [7].
  In order to achieve reuse, Kim et al. [8] invented an HVR/SAF hybrid nonwoven that can be made into reusable incontinence pads. There are several disadvantages to using disposable diapers, incontinence pads, and other absorbent hygiene products. On the one hand, their friction with the skin may develop into irritant contact dermatitis, and eventually lead to skin infection. On the other hand, most of the filling materials are difficult to degrade, which will cause environmental pollution after being discarded.
2. Secondly, some doctors use drugs to treat incontinence. Bliss et al. [9] found an improvement in incontinence by changing the composition and concentration of stools by adding bulking agents to foods. The results of Read et al. [10] showed that loperamide not only improved stool consistency and reduced stool weight but also significantly improved incontinence. Santoro et al. [11] treated incontinence patients with Amitriptyline, and symptoms improved significantly after four weeks of treatment. Medication does not work well for all incontinence patients, and most medications have side effects; therefore, it may not be a good solution for incontinence in the disabled elderly.
3. For patients with severe incontinence, surgical treatment is an option after attempts at nonsurgical treatment have failed. Srinivas et al. proposed in the literature [12] that Sphincter Repair could be used to improve incontinence symptoms for patients with minor sphincter damage; however, this option is not feasible for patients with severe sphincter damage. Later, Lehur et al. [13] removed the damaged sphincter during surgery and implanted an artificial sphincter in the patient’s body, which was effective for severe fecal incontinence. Colquhoun et al. [14] investigated patients after the surgery and found significant improvement in incontinence symptoms and quality of life. However, the risk of wound infection and the high rate of complications associated with any of these procedures have led to these procedures not being widely accepted.

### 1.1. Motivation

**Figure 1:**
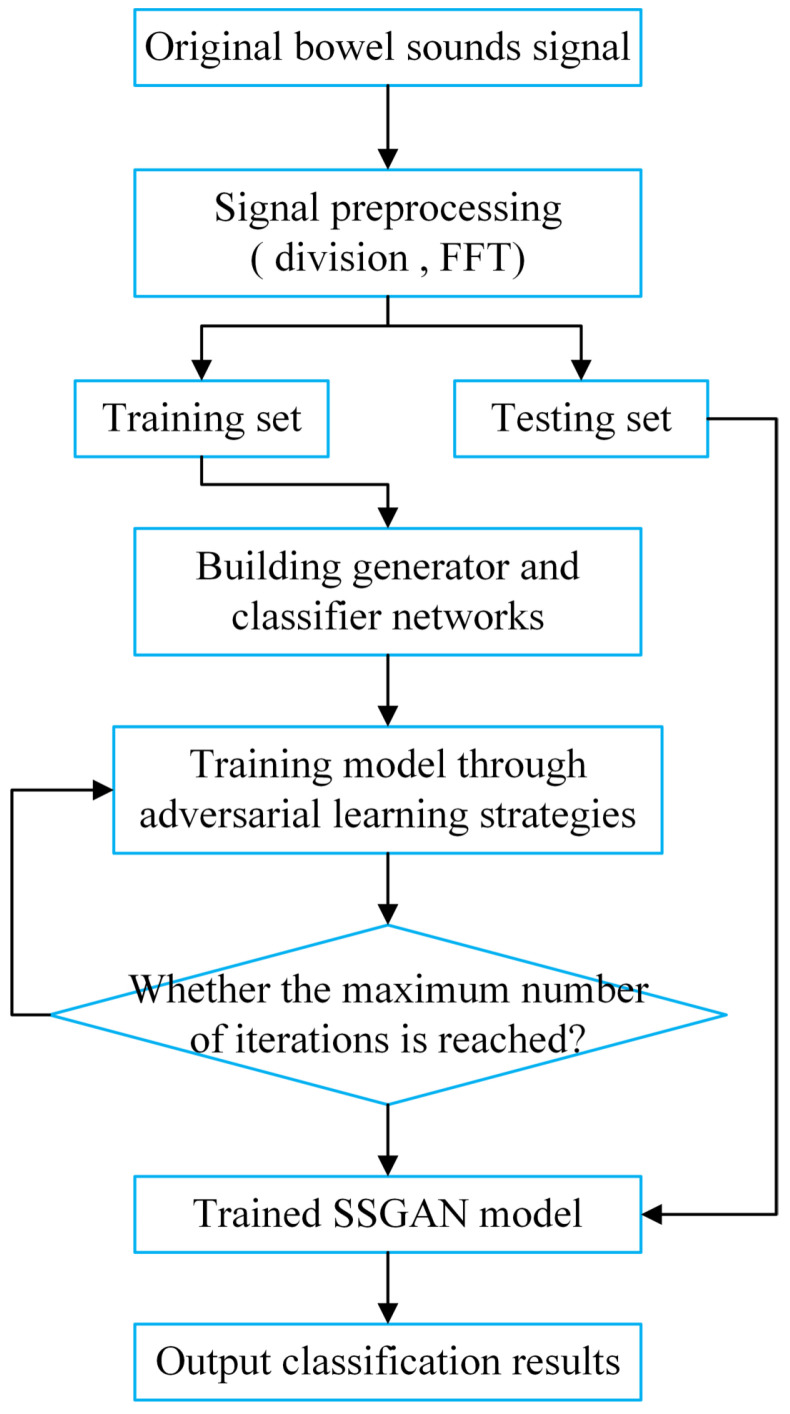
Flowchart of the proposed method.

In the past, when researchers solved the problem of fecal incontinence in elderly disabled patients, almost all of them focused on how to improve the treatment methods after the incontinence, and few studied how to solve the problem before the incontinence. In this paper, we attempted to find a way to predict the time of fecal excretion to predict fecal incontinence. In this paper, a method is proposed to predict the defecation time by monitoring the changes of bowel sound signals before and after defecation, as shown in Figure 1, so as to warn and deal with the defecation behavior of patients in advance, which is expected to greatly reduce the burden of nurses. The defecation pre-warning proposed in this paper refers to accurately predicting the subject’s upcoming defecation behavior within 15 min of defecation.

As a universal phenomenon, bowel sounds have attracted people’s attention for a long time. Farrar et al. [15] were the first to study the connection between bowel sounds and intestinal movement and published relevant articles as early as 1955. Liu et al. [16] proved that bowel sounds are caused by the movement of gas or mixture in the intestinal lumen, in which the sound of gas cracking is the main part.

Bowel sounds were chosen for two reasons. The first reason is that Saito et al. [17], in their study of the mechanism of bowel sounds, found that bowel sounds are produced by the movement of intestinal contents, which is caused by defecation. Therefore, bowel sounds before defecation are different from those during normal periods (except for one hour before and after defecation). Theoretically, we can judge whether the human body has the intention of defecation or not by analyzing the waveform of bowel sounds, which is also proved in the experiment in Section 4. The second reason is that Bampton et al. [18] found that the frequency and amplitude of colonic motility associated with defecation began to increase 1 h before defecation began.

This detection of bowel sounds is non-invasive, low-cost and painless. Dalle et al. [19] proposed a method for the automatic analysis of bowel sound recordings, which measured the frequency of bowel sound in a quiet environment. Most of the energy of bowel sound data was concentrated below 1500 Hz. Ranta et al. [20] further confirmed that the frequency of bowel sound signals is mostly between 100 and 500 Hz, only about 2% exceed 500 Hz and only 0.5% above 1000 Hz.

Du et al. [21] realized low-cost gastrointestinal motion detection by analyzing the waveform and spectrum of bowel sounds. Liatsos et al. [22] recorded the bowel sounds of patients with small-volume ascites and a control group and analyzed them using wavelet transform. They found that bowel sounds were helpful for the diagnosis of small-volume ascites.

Ching et al. [23] used an electronic stethoscope to record the bowel sounds of patients with bowel obstruction, and proved that the characteristics of intestinal sounds may be helpful to determine the location of obstruction through duration, interval between sounds, dominant frequency and peak frequency. With the development of science and technology, methods and equipment for measuring bowel sounds are constantly proposed. Zhang et al. [24] designed an eight-channel data acquisition system based on C8051F340 microcontroller, realizing real-time detection, display and storage of intestinal sounds.

Turk et al. [25] proposed a biosensor-based bowel sound detection system that can reduce environmental noise and transmit data through wireless connection due to the ZigBee module. A team from Tsinghua University in China [26] developed a flexible device for the long-term, real-time monitoring of bowel sounds consisting of an elastic resonator and flexible electronics that can be flexibly attached to the skin surface of the abdomen and bend as the abdomen rises and falls during breathing without distorting the data. These studies demonstrate the medical value and feasibility of bowel sounds.

### 1.2. Contribution

Incontinence is divided into fecal incontinence and urinary incontinence. This paper mainly studies the early warning methods of fecal incontinence (FI). In order to realize the early warning of defecation in the disabled elderly, we study and propose a pre-warning algorithm of defecation in the disabled elderly based on a semi-supervised generative adversarial network.

1. Goodfellow et al. [27] proposed the generative adversarial network (GAN) framework in 2014, including generator G and discriminator D, which is an application of zero-sum game theory. GAN does not need complex Markov chains in the learning process and can easily incorporate the interaction of various factors into the model. Therefore, a variety of GAN technologies are developing rapidly, and GAN can be seen in many fields of research, such as image generation, image restoration, text generation and fault diagnosis [28], and have achieved excellent results. In the development process, Springenberg [29] and Salimans et al. [30] used GAN to complete classification tasks, and Odena [31] proposed a new semi-supervised GAN network (SSGAN) in 2016 that achieved excellent classification results on MNIST data sets.
  At present, SSGAN has particularly outstanding performance in fault diagnosis [32], image classification [33] and other fields. In this paper, we propose a defection pre-warning algorithm based on a semi-supervised generative adversarial network (SSGAN) for the disabled elderly, which can solve the incontinence nursing problem of the disabled elderly without making them feel pain. This is the first application of SSGAN in the field of health care for the disabled elderly. In the experimental process, we found that the trained early warning model could classify intestinal sounds with 94.4% accuracy, which is expected to be applied in practical scenarios to assist doctors in treatment and reduce the burden of nurses.
2. We integrated a physiological signal acquisition system, which can collect three kinds of signals, such as bowel sounds, gastric electrical signals and ECG signals. In order to ensure the authenticity and authority of the experimental data, we collected data from Beijing Bo’ai Hospital and South China University of Technology. The former is affiliated with the China Rehabilitation Research Center. All data were collected with the knowledge and consent of the volunteers.

## 2. Physiological Signal Acquisition

### 2.1. Composition of the Physiological Signal Acquisition System

**Figure 2:**
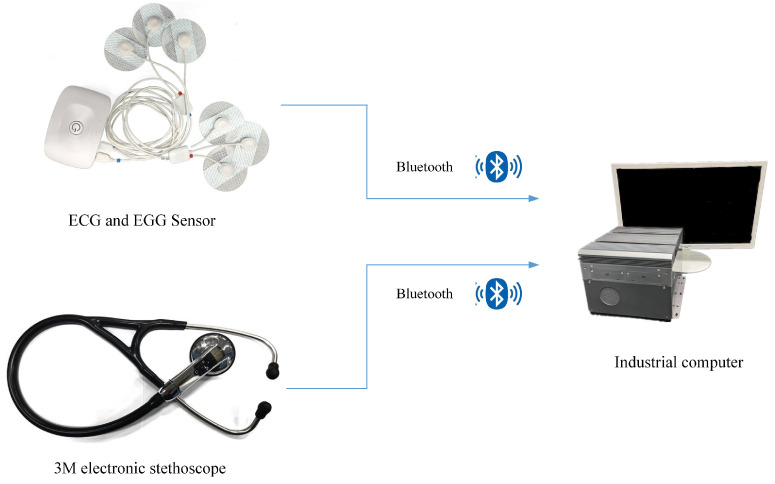
Physiological signal acquisition system.

Figure 2 shows the physiological signal acquisition system, which mainly consists of an electronic stethoscope, gastro-electric sensor, cardio-electric sensor and industrial control computer.

**Figure 3:**
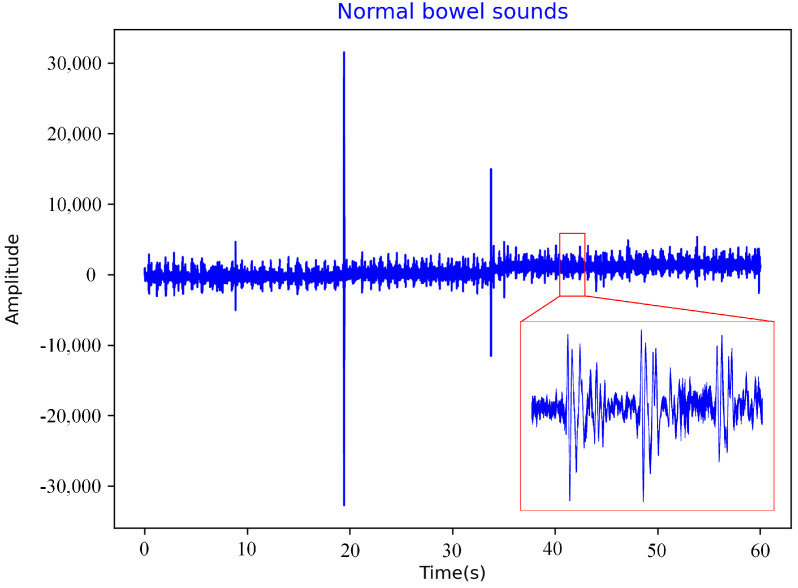
Normal bowel sounds.

**Figure 4:**
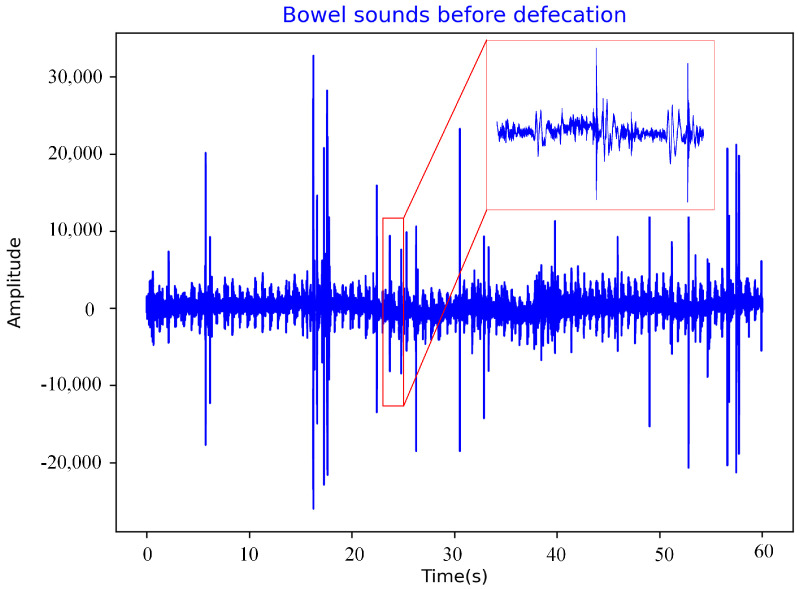
Bowel sounds before defecation.

The physiological signal acquisition system can collect bowel sounds, gastric electrical signals, and ECG signals. The sensor for collecting bowel sounds is a Littmann 3200 electronic stethoscope (hereinafter referred to as the 3M stethoscope) manufactured by the 3M company (Minnesota Mining and Manufacturing corporation, Maplewood, MN, USA). It is a piezoelectric stethoscope with a sampling frequency of 4 kHz, which can convert sound energy into electrical signals and record them [34] as shown in Figure 3 and Figure 4. Most of the energy of intestinal sounds is below 1500 Hz [19].

In this paper, the membrane filter built into 3M stethoscope was selected, which can amplify the sound of 20–2000 Hz and strengthen the sound of 100–500 Hz. This is consistent with the frequency characteristics of bowel sounds and helps to collect bowel sounds. The sensors for collecting gastric and ECG signals are Biosignalsplux’s four-channel sensors with a sampling frequency of 3 kHz. Each sensor collects EMG signals through three electrodes attached to the human body and transmits the collected information to an industrial control computer via Bluetooth. The three electrodes are positive, negative and reference electrodes, respectively.

Industrial control computers are used to process the collected physiological signals. The data processing program was developed by Microsoft Visual Studio, which can realize automatic data collection and saving. After the pre-warning model is trained, the physiological data collected can be input into the model in real-time for classification.

### 2.2. Details of Signal Acquisition

Among the three collected signals, the bowel sound data was selected as the experimental data in this study, and other data will be studied in the future.

The abdomen of the human body is not a hollow cavity but is filled with soft tissue. The density of these soft tissues is also not completely uniform. Therefore, we considered two problems when collecting bowel sounds. One is that there is a sound delay between the source of bowel sounds and the sensor, and the other is that bowel sounds may be absorbed by soft tissue, thus, affecting the waveform.

Peter et al. [35] mentioned that the velocity of sound waves through soft tissues is about 1500 m/s. The distance between any location on the abdominal surface and the source of bowel sounds is relatively small for this number, meaning there is little sound delay. Dimoulas et al. [36] found, on the basis of Craine et al. [37] and Ranta et al. [38], that the absorption of intestinal sounds by soft tissues in the abdomen is negligible. With the above two problems eliminated, the acquisition of the original intestinal sound signal becomes feasible.

**Figure 5:**
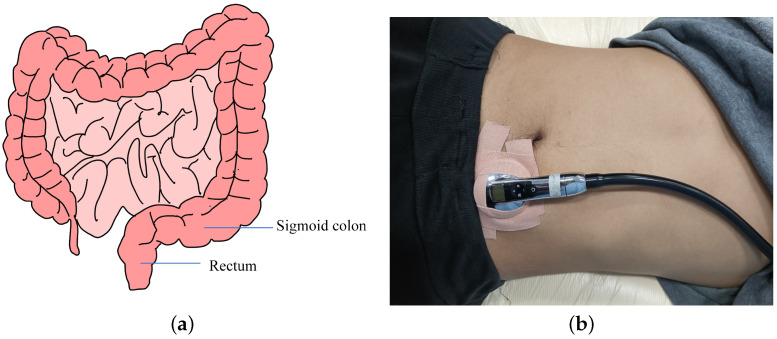
(**a**) Schematic diagram of the intestinal tract. (**b**) Schematic diagram of bowel sound signal collection.

The period from the time when rectal dilatation exceeds the threshold to the time when defecation begins is the time period for collecting fecal bowel sounds in this study. The bowel sounds are caused by compressed feces and bubbles bursting between the contents of the rectum; thus, the stethoscope should collect data close to the rectum. After turning into feces, food debris moves through the large intestine at a rate of about 5 cm per hour and finally slowly enters the rectum from the sigmoid colon, which is on the left side of the rectum [39] as shown in Figure 5a. The stethoscope is located to the left of the navel, as shown in Figure 5b, near both the rectum and sigmoid colon, and is better able to collect bowel sounds.

The original bowel sounds signal is a one-dimensional time series. With the development of bowel sound collection and recording equipment, some common speech signal processing and fault diagnosis processing methods have been applied to bowel sounds processing. As early as 1975, Dalle et al. [19] used fast Fourier transform to analyze intestinal sounds by computer. Subsequently, Dimoulas et al. [40] adopted wavelet transform in their analysis, while Ulusar et al. [41] adopted discrete Fourier transform.

**Figure 6:**
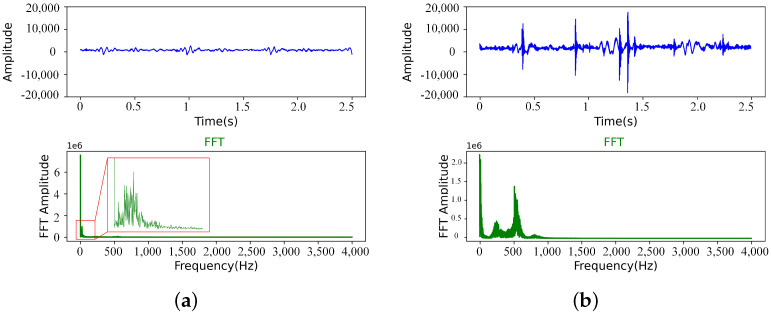
(**a**) Normal bowel sounds (After segmentation). (**b**) Bowel sounds before defecation (After segmentation).

Gary et al. [34] summarized many signal processing methods for automatic detection of bowel sounds by extracting features from acoustic signals, which can be roughly divided into three parts: time domain, frequency domain and time-frequency domain. In this paper, the 3M stethoscope was used to filter the intestinal sound signal when recording data. In the experiment, we found that the classification effect obtained by training network after fast Fourier transform (FFT) of the collected intestinal sound is better. Therefore, the experimental data pretreatment method in this paper is to segment the data and then perform fast Fourier transform as shown in Figure 6.

## 3. Proposed Algorithm

### 3.1. Generative Adversarial Network

Goodfellow et al. [27] proposed the generative adversarial network (GAN) framework in 2014, including generator *G* and discriminator *D*, which is an application of zero-sum game theory. In the process of training the network, random noise is fed into generator G to produce output that looks like real data or a picture. The task of the discriminator is to distinguish the generated sample from the real sample and feed the discriminant result back to the generator.

The training process of GAN is to use the discriminant results of the discriminator to guide the training of the generator, whose purpose is to learn the distribution of real data and generate real data or images, so that *D* cannot judge whether the input is generated data or real data. The purpose of the discriminator is to continuously improve the discriminant ability to separate the generated data from the real data. This is the basic principle of GAN, and the above process can be expressed mathematically as

(1) $$\min_{G}\max_{D}V\left(D,G\right)=E_{x∼P_{data}\left(x\right)}\left[logD\left(x\right)\right]+E_{z∼P_{z}\left(z\right)}\left[log\left(1-D\left(G\left(z\right)\right)\right)\right]$$

where *x* is real data; *z* is random noise of the input; $P_{data}\left(x\right)$ represents the distribution of real data; $P_{z}\left(z\right)$ represents the distribution of random noise; E(⋯) represents their respective mathematical expectations; G(z) represents the data generated by the generator; and D(⋯) represents the probability that the data input to the discriminator comes from real data and is a real number [0, 1].

**Figure 7:**
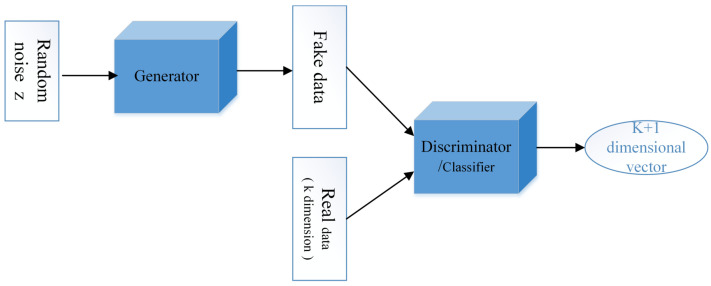
Basic structure of SSGAN.

In the development of GAN, Springenberg et al. [29] proposed CatGAN in 2016, a method for learning discriminant classifiers from unlabeled or partially labeled data. Salimans et al. [30] improved the training technique of GAN and completed the semi-supervised classification task. Miao et al. [42] proposed an improved GAN network for fault diagnosis of rotating machinery, which achieved good performance with limited data. Odena, A [31] proposed a new semi-supervised GAN network (SSGAN) in 2016, changing the output of discriminator *D* to full-connection layer output and the number of neurons from 1 to K + 1. K represents the number of data types in the training set, and the extra 1 corresponds to the data generated by generator *G*. In other words, discriminator *D* is changed into classifier *C* as shown in Figure 7.

SSGAN has had some successful applications in image processing and signal processing. Han et al. [43] proposed a semi-supervised generation framework (SSGF), which effectively solved the problem of scene classification of high-resolution images lacking annotations. Liang et al. [44] proposed an intelligent fault diagnosis method based on SGAN and wavelet transform, which achieved a good fault diagnosis effect in the case of a small number of labeled samples.

Trinh et al. [45] proposed a classification method for pathological signals based on SSGAN, which treated the generated data of generator *G* as unlabeled data and modified the discriminator into a classifier, thus, solving the two problems of insufficient data and data classification. Li et al. [46] proposed a two-stage transmission adversarial network (TSTAN) based on adversarial learning for multi-fault detection of rotating machinery, which could classify not only known faults but also unlabeled new faults and handle tasks of new faults in the target domain.

### 3.2. Framework of the Proposed Method

In this paper, a defecation pre-warning algorithm based on a semi-supervised generative adversarial network (SSGAN) is proposed for the disabled elderly in bed. A real-time collection of bowel sounds was used to determine whether the volunteers wanted to defecate. The data collected by the stethoscope were divided into labeled training samples and unlabeled test samples. We used the labels 0 and 1, where 0 means “do not want to defecate” and 1 means “want to defecate”.

“Do not want to defecate data” refers to the bowel sounds collected during the time period when the subject has no desire to defecate after eating for at least one hour, labeled 0. If the subject felt a desire to defecate and successfully defecated within 15 min of collecting the bowel sound signal, the data were considered as “want to defecate” and labeled as 1. Bowel sounds in the training set and test set were not duplicated, and all samples in the test set were unlabeled. The SSGAN structure designed in this study consists of two parts—namely, generator *G* and classifier *C*.

**Figure 8:**
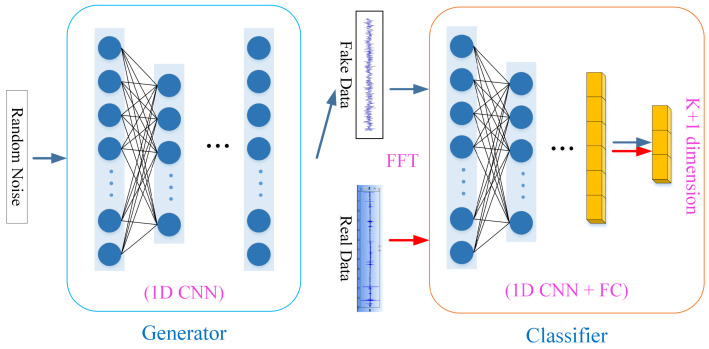
Network structure diagram.

The detailed structure and parameters of SSGAN are shown in Figure 8, Table 1 and Table 2, respectively. The input of the generator network is random noise and the output is fake data whose shape is the same as the real data after preprocessing. The internal network layer is one-dimensional convolutional neural network (1D CNN), which is different from the two-dimensional neural network commonly used in image processing. The one-dimensional convolutional neural network can directly process one-dimensional time series without using wavelet transform and other means into two-dimensional time-frequency images.

Classifier C is improved from discriminator D in original GAN. The last layer of D is sigmod function, which outputs the numbers from 0 to 1 and can only distinguish true and false data. The last layer of C is the Softmax function, and the full connection layer has K + 1 neurons. In this paper, K is 2—that is, the classifier divides the data into three categories (namely, the fake data generated by the generator with and without defecation tendency).

#### 3.2.1. Loss Function

Compared with the original GAN, the generator of SSGAN used in this paper has not changed; however, discriminator D is changed into classifier C. Therefore, this paper only needs to define the loss function of classifier C, which consists of the loss of supervised learning and the loss of unsupervised learning [47]. The formula is as follows,

(2) $$Loss=L_{1}+L_{2}$$

(3) $$L_{1}=-E_{x,y∼P_{data(x,y)}}log\left[P_{model}\left(y|x,y<K+1\right)\right]$$

(4) $$L_{2}=-\left\{E_{x∼P_{data(x)}}log\left[1-P_{model}\left(y=K+1|x\right)\right]+E_{z∼P_{z}}log\left[P_{model}\left(y=K+1|x\right)\right]\right\}$$

where $L_{1}$ represents the loss of supervised learning; $L_{2}$ represents the loss of unsupervised learning; $P_{data}$ represents the distribution of real data and labels; and $P_{model}$ represents the probability of data being identified as each category. The function used is the cross entropy loss function, which is expanded as (5),

(5) $$p\left(y=i\right)=\left[\begin{array}{c} p(y=1)\\ p(y=2)\\ p(y=3)\\ \cdots \\ p(y=K+1) \end{array}\right]=\frac{1}{\sum_{1}^{K+1}e^{x_{i}}}\left[\begin{array}{c} e^{x_{1}}\\ e^{x_{2}}\\ e^{x_{3}}\\ \cdots \\ e^{x_{K+1}} \end{array}\right],$$

which $P_{model}\left(y<K+1\right)$ represents the probability that data will be classified into class *K* after passing through softmax layer. The probability that the first class *K* are all true data. $P_{model}\left(y=K+1\right)$ represents the probability of categorization as false data. Since there are only two types of labels for real data, *K* = 2.

#### 3.2.2. Hyperparameter Configuration

The hyperparameters will affect not only the training time of the model but also the classification accuracy of the model [17]. Choosing an appropriate hyperparameter can make the network have better performance. The sampling frequency of the 3M stethoscope is 4 kHz, and it is collected for 60 s at a time; thus, the original intestinal sound signal is a one-dimensional time series composed of 240,000 points. The input data length of a one-dimensional convolutional neural network is an important parameter. Many times of experimental training experience tells us that direct use of original signal input will bring a great deal of calculation, however, will also make the model classification effect worse.

However, if the raw data is divided too finely to show the complete information of a bowel sound, the classification results will be meaningless. Finally, we found that the best results were obtained by dividing the data into a size of 10,000. See Table 1 and Table 2 for other parameters. The network was trained in the PyTorch framework with a learning rate set to 0.002 and iterating over 100 epochs. Considering the large amount of intestinal sound data, a small batch size of 10 was used. Multiple experiments have proved that this setting has the best classification effect.

#### 3.2.3. Network Training

The generator is built from the architecture shown in Figure 8 and Table 1.The random noise input to the generator has a shape of 10,000 × 1, passing through the full connection layer 160,000 × 1. Use the view function to reshape to (2500, 1, 64). Two up-sampling layers are then used to increase (2500, 1, 64) to (10,000, 1, 64). After each up-sampling layer, we apply the convolution layer with a convolution kernel of 3 and step of 1 as well as a batch normalization layer with a momentum of 0.8, and finally we generate the fake data shape of (10,000, 1, 1).

The classifier is built from the architecture shown in Figure 8 and Table 2. The shape of the data input to the classifier is (10,000, 1, 1). We apply the convolution layer with four convolution kernels of eight steps and four steps. After each convolution layer, we apply LeakyReLU, whose alpha value is 0.2.

The output of the last convolution layer is then fed into the full connection layer. The activation function of the final classification layer is Softmax, which has three neurons. The data were divided into three categories: fake data, with or without defecation tendency.

## 4. Experiments and Discussion

### 4.1. Bowel Sounds Dataset

To verify the effectiveness of the proposed method, we constructed a bowel sounds dataset. The data were collected at Beijing Bo’ai Hospital and South China University of Technology, and all volunteers were informed and consented to our work. The data set included bowel sounds from 15 volunteers aged 23 to 70 years. They were not taking any drugs that affected bowel motility in the recent period of data collection. Before collecting the bowel sounds data, the hair on the patient’s abdomen was cleaned and they laid down in a quiet environment to avoid noise and environmental noise caused by hair friction.

A 3M electronic stethoscope was fixed on the lower right side of the volunteers’ abdomen with medical tape Figure 5b. The signal collected by the stethoscope was recorded as a WAV file. As mentioned above, the frequency of bowel sounds is mostly below 1 kHz, and the sampling frequency of the stethoscope was 4 kHz, which can meet these requirements.

The sampling frequency of the 3M stethoscope was 4 kHz, and the time of one sampling was 60 s. The length of data collected at one time was 240,000 × 1. If such long original data are directly placed into a one-dimensional convolutional neural network for training, many convolutional layers are required, resulting in a slow training speed and slow network convergence. In this experiment, 240,000 1 bowel sounds data of 1 min were segmented, and the size of the segmented data was 10,000 and labeled. Normal bowel sounds were labeled 0, and bowel sounds before defecation were labeled 1.

### 4.2. Compared Approaches

In order to verify the effectiveness of the proposed method, three typical methods are selected for comparison. Trinh et al. [45] used CNN to compare the SSGAN proposed when classifying pathological speech. The CNN used in this paper has the same structure as the classifier in the proposed SSGAN to ensure that the performance of CNN is directly comparable with that of the proposed method. Zheng et al. [48] used CNN+BiGRU to classify body sounds. The simple structure of this network makes it universal and can be applied to other audio data sets, such as breath sounds and heart sounds. The third comparison method is LSTM, which belongs to the recurrent neural network with the second method. The difference is that LSTM networks combine short-term and long-term memory. Zheng et al. [48] used it to compare with CNN+BiGRU.

### 4.3. Experimental Result

To evaluate the performance of the proposed SSGAN method, a set of comparative experiments were designed. Six different test sets were placed into four models for classification. The data was processed with FFT before entering the network. The Adam optimization method was adopted. In network training, the batch size was set to 10. There were 200 data samples in each test set. Data before defecation was harder to collect, and thus there were fewer defecation data in the test set—only 60. The classification accuracy of the six test sets in the four models is shown in Table 3.

**Figure 9:**
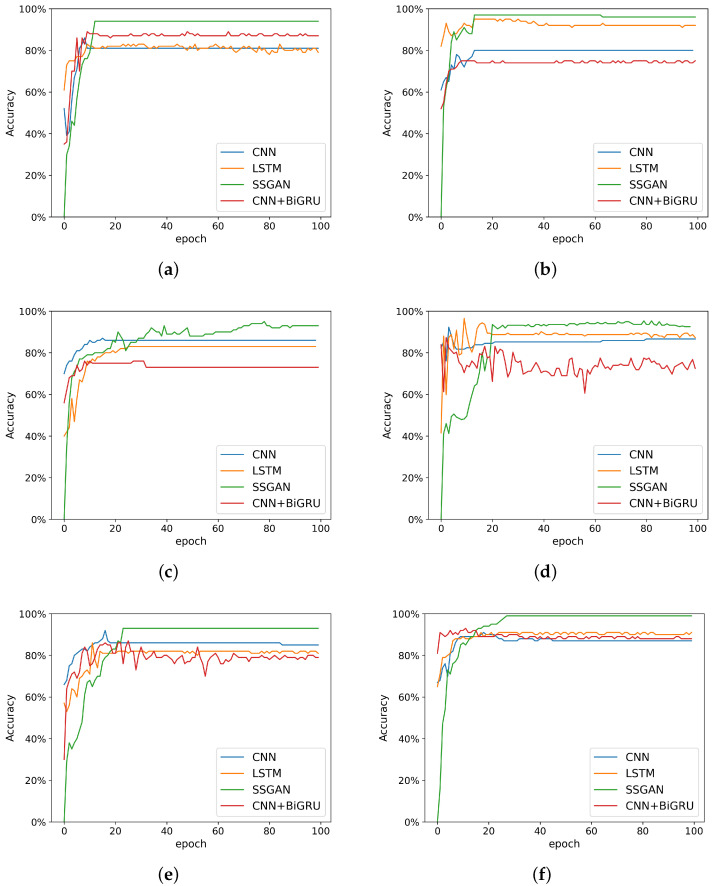
Accuracy of the test sets. (**a**) Accuracy of test set A; (**b**) Accuracy of test set B; (**c**) Accuracy of test set C; (**d**) Accuracy of test set D; (**e**) Accuracy of test set E; (**f**) Accuracy of test set F.

It can be seen that on different test sets, the classification effect of the proposed method is superior to the three comparison methods. Our results confirm the feasibility of using SSGAN classification method. Figure 9 and Table 3 show the experimental results. It can be seen that the average accuracy of the three comparison methods was lower than that of SSGAN. In other words, SSGAN had better performance. The average accuracy of this method for all classification tasks was about 94.4%.

It should be noted that SSGAN’s classification accuracy was 0 at the beginning. This is because it classifies all the data as fake data generated by the generator. It can be seen that the classification accuracy of the other three methods is not ideal in the training process. The proposed method based on SSGAN had high classification accuracy and stable effect. Since the test set in this paper is an unbalanced data set, the specificity and sensitivity indexes of the method proposed in this paper were calculated, and the results are shown in Table 4.

(6) $$Accuracy=\frac{TP+TN}{TP+FP+TN+FN}$$

(7) $$Spc=\frac{TN}{TN+FP}$$

(8) $$Sen=\frac{TP}{TP+FN}$$

where TP is true positive, TN is true negative, FP is false positive, FN is false negative, Spc is specificity and Sen is sensitivity.

As shown in Table 4, the average specificity was 95.1% and the average sensitivity was 92.7%. According to these indexes, the proposed method has good classification performance. Since the goal of defecation prediction is to reduce the complicated cleaning work after defecation, the sensitivity of the model is important. In the test set, 92.7% of bowel sounds were correctly classified, indicating an effective prediction of defecation.

### 4.4. Discussion

The experimental results show that the proposed method can effectively classify two bowel sounds with or without defecation tendency. A 3M electronic stethoscope was used to collect data in Beijing Bo’ai Hospital. In fact, the bowel sounds classification network proposed is based on SSGAN, which can learn the characteristics of the data of different labels. A warning can be given when bowel sounds are detected in the application with a category of 1 (with defecation tendency). This has broad application prospects in the field of health care for the disabled elderly.

Although the method proposed in this paper achieved a good classification effect in the experiment, it has some limitations. First, the signal must be acquired without noise interference, as noise can degrade the data quality. Second, the amount of bowel sounds before defecation is not particularly sufficient. In the future, we plan to improve the above two aspects by using filtering and data enhancement methods. At present, using deep learning to enhance small sample data is a popular direction.

## 5. Conclusions

Incontinence among the disabled elderly is one of the challenges of population aging. In the health care of the disabled elderly, it is necessary to solve the problem of incontinence. In this paper, an early warning method of defecation based on SSGAN was proposed. By using the proposed method to process data and train networks, a strong robustness classification network was obtained. Based on the proposed method, we conducted a comparative experiment on the classification effect of different bowel sound datasets. The experimental results showed that the proposed method effectively classified bowel sounds as with or without defecation tendency.

The proposed method is superior to the comparison method in classification accuracy. This shows that the method proposed in this paper is correct and feasible for the pre-warning of defecation in the disabled elderly, which is meaningful for their health care. The method proposed in this paper can effectively aid in the incontinence problem of the disabled elderly. Moreover, real-time monitoring can be performed, which provides a good possibility for reducing the burden of nurses and assisting doctors in treatment.

## Figures and Tables

**Table 1:** Detailed parameters for generator G.

Layer Type	Activation Function	Kernel Size	Stride	Padding	Output Size
Input	/	/	/	/	10,000 × 1 × 1
FC	/	/	/	/	2500 × 1 × 64
Upsampling	/	/	/	/	5000 × 1 × 64
Convld_1	ReLU	3	1	1	5000 × 1 × 64
BatchNorm	/	/	/	/	/
Upsampling	/	/	/	/	10,000 × 1 × 64
Convld_2	ReLU	3	1	1	10,000 × 1 × 32
BatchNorm	/	/	/	/	/
Convld_3	Tanh	3	1	1	10,000 × 1 × 1

*FC* is fully connected layer, *Convld* is convolutional Layer, and *BatchNorm* is Batch Normalization.

**Table 2:** Detailed parameters for classifier C.

Layer Type	Activation Function	Kernel Size	Stride	Padding	Output Size
Input	/	/	/	/	10,000 × 1 × 1
Convld_1	LeakyReLU	8	4	2	2500 × 1 × 64
Convld_2	LeakyReLU	8	4	0	624 × 1 × 64
Convld_3	LeakyReLU	8	4	0	155 × 1 × 64
Convld_4	LeakyReLU	8	4	0	37 × 1 × 1
FC	Softmax	/	/	/	3

*FC* is fully connected layer, *Convld* is convolutional Layer.

**Table 3:** Accuracy of different methods in six test sets.

Tasks	LSTM	CNN	CNN + BiGRU	SSGAN
A	79.5%	81.5%	87.0%	92.0%
B	92.0%	80.5%	74.5%	96.0%
C	82.5%	86.0%	73.0%	93.5%
D	86.5%	83.0%	77.0%	92.5%
E	82.0%	85.5%	79.5%	93.5%
F	91.5%	87.0%	88.0%	99.0%
Average	85.7%	83.9%	79.8%	94.4%

**Table 4:** The specificity and sensitivity of SSGAN.

Tasks	TP	TN	FP	FN	Specificity	Sensitivity
A	55	129	11	5	92.1%	91.7%
B	56	136	4	4	97.1%	93.3%
C	53	134	6	7	95.7%	88.3%
D	56	129	11	4	92.1%	93.3%
E	55	132	8	5	94.3%	91.7%
F	59	139	1	1	99.3%	98.3%
Average	55.6	133.1	6.8	4.3	95.1%	92.7%

*TP* is true positive, *TN* is true negative, *FP* is false positive, *FN* is false negative.

## Data Availability

We created a dataset for this study. Since further research is in progress, we cannot publish the dataset right now.
